# Delaying anterior cruciate ligament reconstruction for more than 3 or 6 months results in lower risk of revision surgery

**DOI:** 10.1186/s10195-024-00759-1

**Published:** 2024-04-18

**Authors:** Helena Amstrup Jensen, Torsten Grønbech Nielsen, Martin Lind

**Affiliations:** 1 Department of Orthopedics, Aarhus University Hospital, Palle Juul-Jensens Boulevard 99, Aarhus N, 8200 Aarhus, Denmark; 2 Department of Physiotherapy and Occupational Therapy, Aarhus University Hospital, Aarhus, Denmark

**Keywords:** ACL, ACL reconstruction, Anterior cruciate ligament reconstruction, Timing, Time from injury to surgery, Revision surgery, ACL reconstruction revision

## Abstract

**Background:**

The objective of this study is to investigate the risk of revision surgery when delaying anterior cruciate ligament reconstruction (ACLR) past 3 months or 6 months after injury.

**Materials and methods:**

A total of 30,280 patients with isolated ACLR were identified in the Danish Knee Ligament Reconstruction Registry and divided into four groups; ACLR < 3 months, > 3 months, < 6 months, or > 6 months after injury. Primary outcome was revision surgery and secondary outcome were objective and subjective clinical outcome. The 2 year relative risk, crude, and adjusted hazard ratio (HR) were calculated.

**Results:**

Comparing ACLR < 3 months to ACLR > 3 months of injury the 2 year relative risk of revision surgery was found to be 1.81 (95% CI 1.46–2.23; *P* < 0.001) with an adjusted hazard ratio (HR) of 1.27 (95% CI 1.12–1.44; *P* < 0.001). Comparing ACLR < 6 months to ACLR > 6 months of injury the 2 year relative risk of revision surgery was found to be 1.61 (95% CI 1.34–1.92; *P* < 0.001) with an adjusted HR of 1.27 (95% CI 1.15–1.40; *P* < 0.001).

**Conclusion:**

The risk of revision ACLR surgery was found to be increased when ACLR was performed within 3 months or 6 months of injury compared with later surgery. The 1 year postoperative objective knee laxity and the subjective patient-related outcome was found to be without a clinically significant difference; however, those with early ACLR (< 3 months or < 6 months) were found to have a higher activity level 1 year postoperatively. The information about increased risk of revision when having early surgery should be informed to patients when deciding timing of ACLR treatment.

*Level of evidence:* II.

## Introduction

The optimal timing of anterior cruciate ligament reconstruction (ACLR) remains uncertain, and there is no consensus on whether early or delayed surgery provides the best outcome.

Early surgery has previously been advised against because of the risk of stiffness and arthrofibrosis [33, 40]. However, more recent studies have reported no significant difference in arthrofibrosis rate when comparing early and late ACLR [6, 15, 26, 45].

Delayed surgery has been known to increase the risk of cartilage and meniscus injury [7, 9, 14, 25, 34] and therefore advised against.

Furthermore, from a social health system perspective, early ACLR is preferable, according to Mather et al. [32] in a cost-effectiveness analysis.

Previous studies investigating the influence of timing of ACLR on revision rates reported no significant difference between early and delayed ACLR [2, 8, 41, 48]. However, a number of more recent studies have reported an increased risk of revision surgery when ACLR was performed early [11, 13, 19, 35, 42], including Ding et al. [13], who reported, based on data from the US integrated healthcare system’s ACLR registry, a significantly higher risk of revision surgery when ACLR was performed within 3 weeks or 3 months of injury compared with more than 9 months after injury. One issue with the comparison of results is the need for consensus on the definition of early and delayed ACLR.

In 2005, the Danish Knee Ligament Reconstruction Register (DKRR) was established as a national clinical database. It contains data from surgeons and patients about all anterior cruciate ligament (ACL) procedures performed in Denmark. This includes data on sagittal and rotatory objective knee laxity, which are not present in the Norwegian and the Swedish National Knee Ligament Registries. Therefore, the DKRR provides an opportunity to investigate revision rates and clinical outcomes for ACLR using a large dataset [28].

The aim of this study is to investigate the result of delaying ACLR past 3 months or 6 months on revision rates and knee stability using data from the DKRR. The tested hypothesis was that surgery within the first 3 months or 6 months of injury increases the risk of revision surgery.

## Materials and methods

This study followed the design of a register-based retrospective comparative cohort study.

### Data source

Data for the present study were extracted from the DKRR as a project specific dataset including the necessary data for the aim of the study. The project and the requested data extraction were approved by the steering comity of the DKRR. National clinical registry studies do not require local ethical committee approval in Denmark.

Data were obtained from the web-based, nationwide, clinical database DKRR [28]. The database includes data from all departments performing ACL procedures in Denmark (both public and private clinics); registration has been compulsory since 2005. The operating surgeon collects data prospectively before surgery, during surgery, and 1 year postoperatively. These data include social security number [29], time of injury, date of surgery, pre- and postsurgery instrumented sagittal knee laxity, and rotatory laxity. Rolimeter or KT-1000 arthrometer tests were used to measure the instrumented sagittal knee laxity between the healthy and the operated knee—measured in mm [4]. In this study, the cohort was grouped in ≤ 2 mm or > 2 mm of side-to-side difference. The pivot shift test was used to measure rotatory laxity of the ACL using a 4-point Likert grading scale, with grade 0 being normal, grade 1 being glide, grade 2 being clunk, and grade 3 being gross [24]. These data were divided into negative pivot shift test results correlating with grade 0 or positive pivot shift test results correlating with grade ≥ 1.

Furthermore, the database contains subjective information from the patient regarding knee function before and 1 year after surgery. Patients independently report these data via the internet using the validated self-assessment scores of the Knee Injury and Osteoarthritis Outcome Score (KOOS) [37] and Tegner activity score [43]. KOOS is a knee-specific score with five subscales, where KOOS4 is a validated outcome using four subscales determining symptoms, pain, sports and recreation, and quality of life, as these are the most responsive subscales from KOOS [18]. Each subscale is rated on a score from 0 to 100, with zero representing extreme knee problems and 100 representing no knee problems [37]. The Tegner activity scale is a knee-specific score from 0 to 10 indicating the highest level of activity that the patient is currently able to perform, with 0 being on sick leave or a disability pension and 10 being involved in competitive sports (soccer, football, and rugby) at an elite national level [43].

### Study population

During the period from 1 July 2005 to 31 December 2018, the authors identified 30,949 patients who had isolated ACLR. Of the 30,949 patients, 669 were excluded because data on the time of injury were missing.

In total, 30,280 patients were included in this study, and all had a minimum follow-up time of 2 years. Based on the time from injury to ACLR, data were divided into 4 groups: 4455 in the < 3 months group, 25,825 in the > 3 months group, 12,518 in the < 6 months group, and 17,762 in the > 6 months group.

### Outcomes

The primary outcome in the present study was ACLR revision, defined as surgical replacement of the primary ACLR graft. Follow-up started on the day of ACLR and ended at the date of revision surgery, death, emigration, or the date of data extraction (31 December 2020).

Secondary outcomes were objective knee laxity and subjective knee function. The objective knee laxity was measured preoperatively and at 1 year follow-up using Rolimeter or KT-1000 arthrometer tests and the pivot shift score as measurements [28]. The patient-reported subjective knee function were measured using KOOS [37] and Tegner activity scores [43] collected preoperatively and at 1 year follow-up.

### Statistics

The total incidence of revision surgery was calculated for each group with a 95% confidence interval (95% CI). Revision rates were estimated as 2 year relative risk (RR) and full follow-up time hazard ratio (HR) with 95% CI to compare those with ACLR < 3 months or < 6 months within injury to those with ACLR > 3 months or > 6 months after injury, respectively. HR was calculated both unadjusted and adjusted for the confounding factors: age, sex (male/female), activity leading to injury (pivoting sport/nonpivoting sport/activity of daily living/traffic/work/unknown), meniscal damage (none/medial/lateral/both), cartilage damage (1–2/3–4/none) and graft choice (hamstring tendon (HT), bone–patella tendon–bone (BTB), and quadriceps tendon–bone or quadriceps tendon (QTB/QT) or other) using Cox regression analysis. Furthermore, the revision rates were compared between study groups (< 3 months versus > 3 months and < 6 months versus > 6 months) using the *χ*^2^ test.

A Kaplan–Meier curve was calculated to illustrate the probability of ACLR revision at different follow-up times.

For instrumented sagittal knee laxity, the mean values of side-to-side difference were calculated in millimeters and compared between groups (< 3 months versus > 3 months and < 6 months versus > 6 months). Furthermore, the proportion of ≤ 2 mm of side-to-side difference was calculated for each group. For the pivot shift test, the proportion of negative tests was calculated for each group. For both pivot shift test and instrumented sagittal knee laxity, proportions were compared between groups (< 3 months versus > 3 months and < 6 months versus > 6 months) using the *χ*^2^ test.

For the KOOS4 score and Tegner activity score, mean values were calculated and compared between groups (< 3 months versus > 3 months and < 6 months versus > 6 months) using the Student *t*-test.

*P* values of < 0.05 were considered to be statistically significant.

Statistical analyses were executed using the software package STATA version v17.0 (StataCorp. 2021. Stata statistical software: Release 17. College Station, TX: StataCorp LLC).

## Results

Demographic data for the total population are presented in Table 1, and patient characteristics for the four groups are presented in Table 2.

**Table 1:** Population demographics

Demographic data	All *n* = 30,280
Age, mean ± standard deviation (SD)	29 ± 11
Sex, female/male %	40/60
Instrumented sagittal knee laxity ≤ 2 mm, *n* (%)	2617 (10.3)
Negative pivot shift test, *n* (%)	3240 (11.3)
Pivoting sport leading to injury, *n* (%)	17,675 (57.3)
Graft choice	
Hamstring tendon, *n* (%)	25,127 (83.2)
Bone–patella tendon–bone, *n* (%)	2865 (9.5)
Quadriceps tendon–bone or quadriceps tendon, *n* (%)	1410 (4.7)
Other, *n* (%)	785 (2.6)
Meniscal damage, *n* (%)	13,316 (43.9)
Cartilage damage, *n* (%)	4500 (17.5)
KOOS4, mean ± SD	55 ± 15.5
Tegner activity score, mean ± SD	3.1 ± 1.9

**Table 2:** Preoperative patient characteristics including data on objective knee laxity and subjective outcomes

Demographic data	< 3 months *n* = 4455	> 3 months *n* = 25,825	*P* value	< 6 months *n *= 12,518	> 6 months *n* = 17,762	*P* value
Age, mean ± SD	27 ± 11	29 ± 11	< 0.001	27 ± 10	30 ± 11	< 0.001
Sex, female/male %	43/57	40/60	<0 .001	40/60	40/60	0.54
Instrumented sagittal knee laxity ≤ 2 mm, *n* (%)	360 (9.8)	2257 (10.4)	0.31	1084 (10.3)	1533 (10.3)	0.98
Instrumented sagittal knee laxity in mm, mean ± SD	4.9 ± 2.0	4.9 ± 2.1	0.09	4.9 ± 1.9	5 ± 2.1	< 0.001
Negative pivot shift test, *n* (%)	380 (9.1)	2860 (11.6)	< 0.001	1239 (10.5)	2001 (11.8)	< 0.001
Pivoting sport leading to injury, *n* (%)	2766 (62.1)	14,909 (57.7)	< 0.001	7954 (63.5)	9721 (54.7)	< 0.001
Graft choice						
Hamstring tendon, *n *(%)	3776 (84.9)	21,351 (82.9)	< 0.001	10,510 (84.1)	14,617 (82.6)	<0 .001
Bone–patella tendon–bone, *n* (%)	323 (7.3)	2542 (9.9)	< 0.001	1015 (8.1)	1850 (10.5)	< 0.001
Quadriceps tendon–bone or quadriceps tendon, *n* (%)	280 (6.3)	1130 (4.4)	< 0.001	713 (5.7)	697 (3.9)	< 0.001
Other, *n* (%)	66 (1.5)	719 (2.8)	< 0.001	254 (2)	531 (3)	< .001
Meniscal damage, *n* (%)	2008 (45.1)	11,308 (43.8)	0.11	5346 (42.7)	7970 (44.9)	< 0.001
Cartilage damage, *n* (%)	529 (11.9)	3971 (15.4)	< 0.001	1556 (12.4)	2944 (16.6)	<0 .001
KOOS4, mean ± SD	51 ± 15.3	56 ± 15.4	< 0.001	53 ± 15.1	56 ± 15.6	<0 .001
Preoperative Tegner activity score, mean ± SD	2.7 ± 2.3	3.0 ± 1.9	<0 .001	2.8 ± 2.1	3.0 ± 1.9	< 0.001
Preinjury Tegner activity score, mean ± SD	6.9 ± 1.9	6.6 ± 1.9	< 0.001	6.9 ± 1.9	6.5 ± 1.9	<0 .001

A significantly lower age, percentage of BTB-grafts, lower percentage of cartilage damage, lower preoperative KOOS4, and higher preinjury Tegner activity score were found in those with ACLR < 3 months or < 6 months of injury. Furthermore, a significantly lower percentage of males was found in those with ACLR < 3 months of injury, and a significantly lower percentage of meniscal damage was found in those with ACLR < 6 months of injury.

### Risk of revision surgery

The total incidence of revision surgery for the group with ACLR < 3 months of injury was found to be 6.8% (95% CI 6.0–7.5%; *P* < 0.001), whereas the total incidence was found to be 5.4% (95% CI 5.2–5.7%; P < 0.001) for the group with ACLR > 3 months after injury. Comparing the groups, a significantly increased risk of revision surgery was found for the group with ACLR < 3 months of injury, with an HR of 1.27 (95% CI 1.12–1.44; P < 0.001) and a 2 year relative risk of 1.81 (95% CI 1.46–2.23; P < 0.001) (Table 3).

**Table 3:** Risk of revision surgery as hazard ratio and 2 year relative risk

Risk of revision	< 3 months versus > 3 months	< 6 months versus > 6 months
Hazard ratio (95% CI)	1.34 (1.18–1.52)	1.47 (1.34–1.62)
Adjusted hazard ratio^a^ (95% CI)	1.27 (1.12–1.44)	1.27 (1.15–1.40)
2 Year relative risk (95% CI)	1.81 (1.46–2.23)	1.61 (1.34–1.92)

^a^Adjusted for age, sex, activity leading to injury, meniscal damage, cartilage damage, and graft choice

The total incidence of revision surgery for the group with ACLR < 6 months of injury was 6.7% (95% CI 6.2–7.1%; *P* < 0.001), whereas the total incidence was found to be 4.9% (95% CI 4.6–5.2%; *P* < 0.001) for the group with ACLR > 6 months after injury. Comparing the groups, the risk of revision surgery was significantly higher when ACLR was performed < 6 months of injury, with an HR of 1.27, (95% CI 1.15–1.40; *P* < 0.001) and a 2 year relative risk of 1.61 (95% CI 1.34–1.92; *P* < 0.001) (Table 3).

**Fig. 1:**
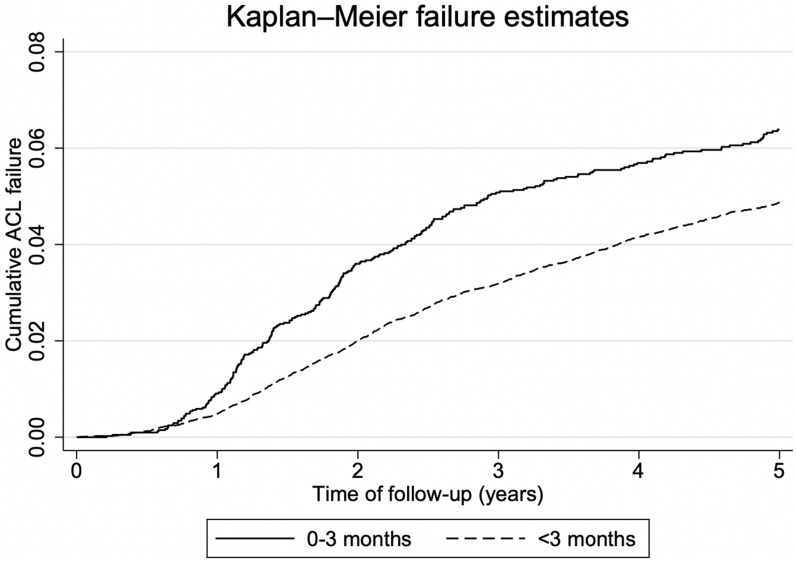
Kaplan–Meier curves showing failure estimates for anterior cruciate ligament reconstruction ≤ 3 months within injury and > 3 months after injury

**Fig. 2:**
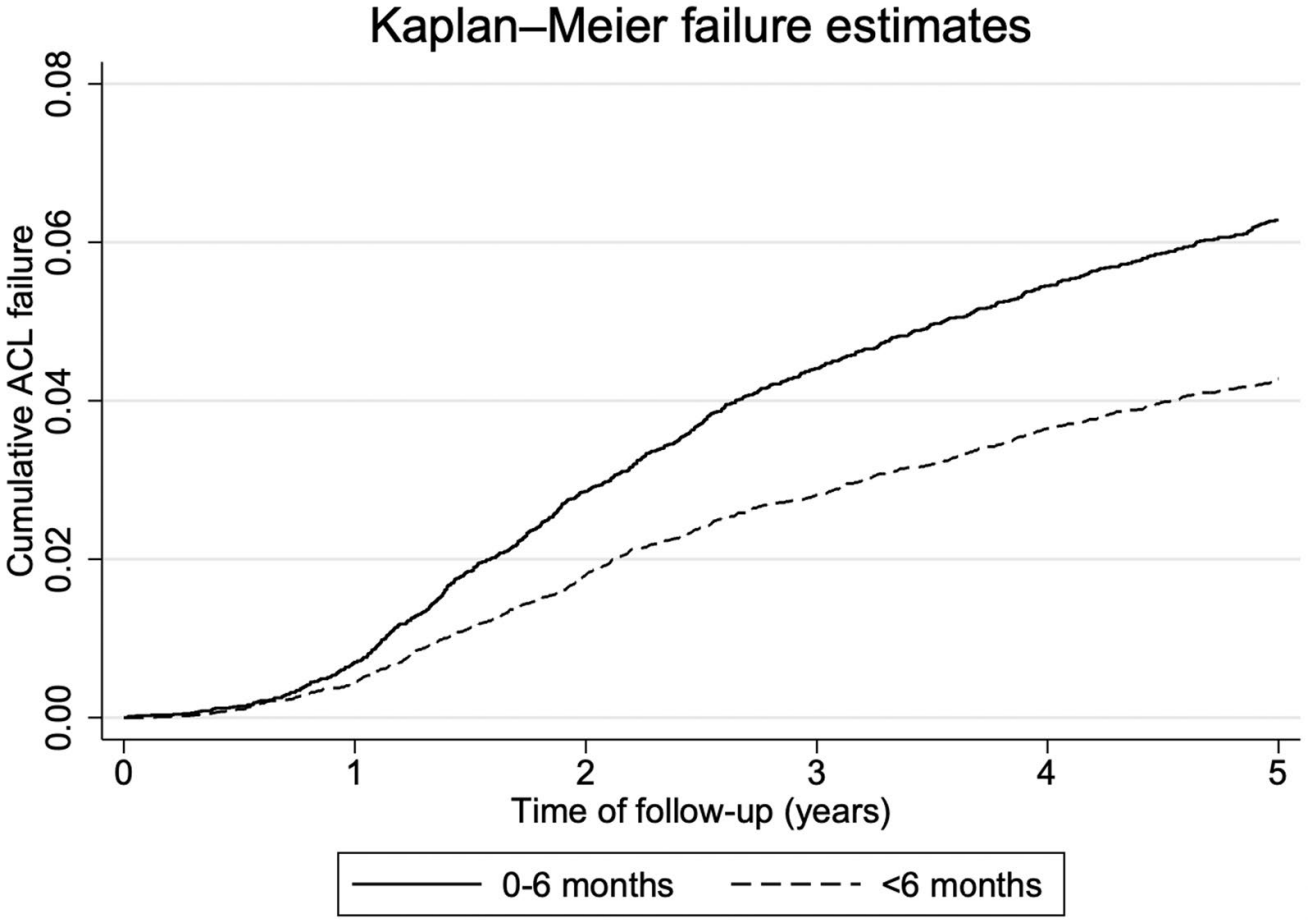
Kaplan–Meier curves showing failure estimates for anterior cruciate ligament reconstruction ≤ 6 months within injury and > 6 months after injury

Cumulated ACL graft failure leading to revision surgery at 5 year follow-up is shown in a Kaplan–Meier curve for both ACLR < 3 months versus > 3 months (Fig. 1) and ACLR < 6 months versus > 6 months (Fig. 2). They show a higher revision rate in the group with early ACLR (< 3 months or < 6 months) after only 1 year.

### Objective knee laxity

Data for instrumented sagittal knee laxity and pivot shift test at 1 year follow-up are shown in Table 4. Assessment of instrumented sagittal knee laxity at 1 year follow-up was performed on 45.7% of patients with ACLR < 3 months, 50.2% of patients with ACLR > 3 months, 50.0% of patients with ACLR < 6 months, and 49.2% of patients with ACLR > 6 months after injury. Pivot shift test was at 1 year follow-up performed on 54.2% of patients with ACLR < 3 months, 58.9% of patients with ACLR > 3 months, 58.4% of patients with ACLR < 6 months, and 58.1% of patients with ACLR > 6 months after injury. ACLR < 3 or < 6 months of injury was found to be associated with a lower objective knee laxity 1-year postoperatively; 86.1% of patients with ACLR < 3 months of injury were found to have an instrumented sagittal knee laxity of ≤ 2 mm in side-to-side difference compared with 82.3% of patients with ACLR > 3 months after injury (p < 0.001), and similar findings were applicable for those with ACLR < 6 months compared with > 6 months after injury. Mean values of side-to-side difference was found to be 1.2 mm and 1.3 mm in those with ACLR < 3 and < 6 months, respectively, compared with 1.4 mm in those with ACLR > 3 or > 6 months. The difference comparing the groups (< 3 versus > 3 months and < 6 versus > 6 months) was found to be statistically significant (*p* < 0.001). Furthermore, a higher proportion of negative pivot shift test results were found for both the early surgery (< 3 months and < 6 months) groups.

**Table 4:** One-year postoperative data on objective knee laxity and subjective outcomes

1 year postoperative follow-up	< 3 months *n* = 2036	> 3 months *n* = 12,975	*P* value	< 6 months *n* = 6261	> 6 months *n* = 8750	*P* value
Instrumented sagittal knee laxity ≤ 2 mm, *n* (%)	1752 (86.1)	10,670 (82.2)	< 0.001	5266 (84.1)	7156 (81.8)	< 0.001
Instrumented sagittal knee laxity in mm, mean ± SD	1.2 ± 1.3	1.4 ± 1.5	<0 .001	1.3 ± 1.4	1.4 ± 1.5	<0 .001

1 year postoperative follow-up	< 3 months *n* = 2416	> 3 months *n* = 15,212	*P* value	< 6 months *n* = 7317	> 6 months *n* = 10,311	*P* value
Negative pivot shift test score, *n* (%)	1979 (85.1)	11,925 (81.1)	< .001	5839 (82.7)	8065 (80.9)	0.003

1 year postoperative follow-up	< 3 months *n* = 1180	> 3 months *n* = 7995	*P* value	< 6 months *n* = 3686	> 6 months *n* = 5489	*P* value
KOOS4, mean ± SD	69 ± 17.2	70 ± 17.4	0.063	69 ± 17.1	70 ± 17.5	0.007
Tegner activity score, mean ± SD	5.4 ± 2.1	4.9 ± 1.9	< .001	5.3 ± 2.1	4.8 ± 1.9	< 0.001

### Subjective outcomes

Data for 1-year postoperative KOOS4 score and Tegner activity score are presented in Table 4. Data regarding KOOS4 score and Tegner activity score were at 1 year follow-up reported by 26.5% of patients with ACLR < 3 months, 29.4% of patients with ACLR < 6 months, and 30.9% of patients with ACLR > 3 months or > 6 months after injury. The mean KOOS4 score was found to be 1 point lower for those with ACLR < 3 or < 6 months of injury at 1 year follow-up; however, it was significantly lower only for those with ACLR < 6 months of injury (*p* = 0.007). A significantly higher Tegner activity score was found for those with ACLR < 3 or < 6 months of injury (*p* < 0.001) at 1 year follow-up.

## Discussion

The primary finding of this study was an increased risk of revision surgery when ACLR was performed within 3 or 6 months of injury relative to ACLR performed later.

This study found the incidence of revision surgery to be increased by 25.9%, from 5.4% when ACLR was performed > 3 months after injury to 6.8% when ACLR was performed < 3 months of injury and 36.7%, from 4.9% when ACLR was performed > 6 months after injury to 6.7% when ACLR was performed < 6 months of injury. These findings could be argued to be of clinical relevance.

Early ACLR is more often offered to younger patients, and as young age is a known independent risk factor of revision surgery [16, 22, 31, 46, 47] this could be a reason for early ACLR having a higher risk of revision surgery. In this study, the groups with early ACLR (< 3 months or < 6 months) were found to be significantly younger than those with ACLR performed later (> 3 months or > 6 months); however, the risk of revision surgery was still found to be significantly increased in those with ACLR within 3 or 6 months of injury after adjusting for age.

In the present study, HT autografts were found to be the most widely used ACLR graft and used significantly more often in those with ACLR < 3 months or < 6 months of injury. More studies have reported that patients having ACLR with HT autografts have a slightly greater risk of revision surgery compared with patients treated with BTB autograft [3, 39]. Furthermore, Runer et al. [38] reported a higher risk of revision surgery in patients treated with HT autograft compared with patients treated with QT autograft; however, this was activity dependent. These results give an indication that graft choice may also contribute to the observed increased risk of revision surgery when ACLR is performed < 3 months or < 6 months of injury, although when adjusting for graft choice, the revision risk was still found to be significantly greater when ACLR was performed early (< 3 months or < 6 months).

The objective knee laxity was found to be statistically greater in the groups with ACLR > 3 months or > 6 months after injury. However, the difference in mean values of side-to-side difference was found to be no more than 0.2 mm comparing those with ACLR < 3 and > 3 months and no more than 0.1 mm comparing those with ACLR < 6 and > 6 months. Magnussen et. al. [30] reported an anterior laxity of up to 6 mm to be without clinical relevance, and the findings of this study must be considered to be without any clinical significance. The observations of this study conflicts with recent results in literature, as Vermeijden et al. [44], in a systematic review and meta-analysis, reported no difference in instrumented laxity when comparing ACLR within 3 or 6 weeks of injury with ACLR later than 3 or 6 weeks after injury, respectively. However, other studies [1, 5] have found similar results to the present study regarding instrumented laxity. A possible reason for these conflicting results could be the lack of consensus on the definition of early and delayed ACLR. This complicates the comparison of the study results.

The KOOS4 score at 1 year follow-up was found to be 1 point lower in the groups with ACLR < 3 months or < 6 months of injury. These findings were found to be significant for those with ACLR < 6 months of injury. However, as the minimal clinically important change in the KOOS scores is considered to be 8–10 points [37], the clinical significance of the findings of this study is probably low.

This study found a significantly higher Tegner activity score in those with early ACLR (< 3 months or < 6 months), both at preinjury and at 1 year follow-up. This represents a greater preinjury and postoperative sport activity in this group, although the difference was found to be no more than 0.5 levels. These findings are comparable to previous study results [12, 17, 23], including Ferguson et. al. [17], who in a systematic review reported a higher Tegner activity score in those with ACLR received early (< 3 weeks). This could be another reason for early ACLR being associated with an increased risk of revision surgery, as Wiggins et al. [47] in a systematic review and meta-analysis reported a return to a high level of activity as a risk factor of secondary ACL injury, as well as Grindem et al. [20], who reported a return to a preinjury (high) level of sports within 9 months of ACLR leading to a higher risk of reinjury.

Patients with delayed ACLR might be better psychologically and physically adjusted to an injured knee [11, 42], which might be another potential reason for increased risk of revision surgery for those with early ACLR. In the present study the preoperative KOOS4 scores and knee laxity at 1 year follow-up were found to be greater in the groups with ACLR > 3 months or > 6 months after injury. This could indicate a better preoperative rehabilitation level and better coping with the ACL injury in these patients. Furthermore, this might result in a more realistic postoperative activity level, which is reflected by a lower 1 year postoperative Tegner activity score in those with delayed ACLR (> 3 months or > 6 months).

### Perspective

The findings of the present study confirm the results reported by other studies [11, 13, 19, 35, 42]. Though good safety of early ACLR regarding range of motion and knee stability have been documented [10, 21, 27], the present study adds to body of evidence that early ACLR is associates with higher risk of later revision. This risk should be informed to patients during the ACL injury treatment decision process so they are informed about this risk if choosing early surgery as possible treatment.

In Scandinavia young and active patients who want to return to pivoting sports are recommended early ACLR. This indicates the still lacking consensus on the optimal timing of ACLR.

### Strengths and weaknesses

This study included a large cohort (*n* = 30,280), exclusively with primary ACLRs and no multiligament procedures. Data were collected over a period of 13 years throughout Denmark.

The risk of information bias is limited, as data are collected prospectively and registration of ACLR is independent of registration of a later revision surgery. However, in many cases, the objective examination was performed by the operating surgeon, which could lead to some information bias regarding objective knee laxity.

A limitation of this study was the low completeness of data regarding objective knee laxity and subjective outcomes. Objective knee laxity at 1 year follow-up was assessed in about 50% of all included patients. Approximately 30% of all included patients reported data on subjective outcome using the self-assessment scores. This is a known and expected challenge for all large registry follow-up studies and could potentially lead to selection bias. However, a previous study on the validity of data from the DKRR found no difference in subjective outcome scores between nonresponders and responders [36].

The present study used revision surgery as the primary outcome. This could underestimate the true incidence of ACL graft failure, as patients with clinical ACL failure who did not have revision surgery for various reasons were not included.

Possible confounders (including sex, age, activity leading to injury, meniscal damage, cartilage damage, and graft type) were included in the multivariate analysis; however, there may be residual confounders as compliance and quality of rehabilitation and 2 year postoperative activity level.

## Conclusions

The risk of revision ACLR surgery was found to be increased when ACLR was performed within 3 months or 6 months of injury compared with later surgery. The 1 year postoperative objective knee laxity and the subjective patient-related outcome was found to be without a clinically significant difference; however, those with early ACLR (< 3 months or < 6 months) were found to have a higher activity level 1 year postoperatively. The information about increased risk of revision when having early surgery should be informed to patients when deciding timing of ACLR treatment.

## Data Availability

The data that support the findings of this study are available from the Danish Knee Ligament Reconstruction Register but restrictions apply to the availability of these data, which were used under license for the current study, and so are not publicly available. Data are however available from the authors upon reasonable request with permission of the Danish Knee Ligament Reconstruction Register.

## Abbreviations

ACLR: Anterior cruciate ligament reconstruction
ACL: Anterior cruciate ligament
DKRR: Danish knee ligament reconstruction register
KOOS: Knee Injury and Osteoarthritis Outcome Score
HT: Hamstring tendon
BTB: Bone–patella tendon–bone
QTB: Quadriceps tendon–bone
QT: Quadriceps tendon

## References

[1] Ahn JH, Lee SH (2016). Risk factors for knee instability after anterior cruciate ligament reconstruction. Knee Surg Sports Traumatol Arthrosc.

[2] Andernord D, Björnsson H, Petzold M, Eriksson BI, Forssblad M, Karlsson J (2014). Surgical predictors of early revision surgery after anterior cruciate ligament reconstruction: results from the swedish national knee ligament register on 13,102 patients. Am J Sports Med.

[3] Arida C, Tsikrikas CG, Mastrokalos DS, Panagopoulos A, Vlamis J, Triantafyllopoulos IK (2021). Comparison of bone-patella tendon-bone and four-strand hamstring tendon grafts for anterior cruciate ligament reconstruction: a prospective study. Cureus.

[4] Arneja S, Leith J (2009). Review article: Validity of the KT-1000 knee ligament arthrometer. J Orthop Surg (Hong Kong).

[5] Baba R, Kondo E, Iwasaki K, Joutoku Z, Onodera J, Onodera T (2019). Impact of surgical timing on clinical outcomes in anatomic double-bundle anterior cruciate ligament reconstruction using hamstring tendon autografts. Orthop J Sports Med.

[6] Berbig R, Rillmann P (2000). Timing of the surgery of rupture of the anterior cruciate ligament. effects of acute or delayed surgery on arthrofibrosis rate and work disability. Unfallchirurg.

[7] Brambilla L, Pulici L, Carimati G, Quaglia A, Prospero E, Bait C (2015). Prevalence of associated lesions in anterior cruciate ligament reconstruction: correlation with surgical timing and with patient age, sex, and body mass index. Am J Sports Med.

[8] Brown M, Hurlburt GA, Koenig ZA, Richards D (2022). The multivariate relationship between primary anterior cruciate ligament reconstruction timing and revision rates: A 10-year analysis. Cureus.

[9] Cance N, Erard J, Shatrov J, Fournier G, Gunst S, Martin GL (2023). Delaying anterior cruciate ligament reconstruction increases the rate and severity of medial chondral injuries. Bone Joint J.

[10] Chua K, Kang JBY, Fook-Chong S, Tan AHC (2021). Anterior cruciate ligament surgery performed less than 3 weeks after injury is not inferior to delayed surgery. J Knee Surg.

[11] Cristiani R, Forssblad M, Edman G, Eriksson K, Stålman A (2021). Age, time from injury to surgery and quadriceps strength affect the risk of revision surgery after primary ACL reconstruction. Knee Surg Sports Traumatol Arthrosc.

[12] de Valk EJ, Moen MH, Winters M, Bakker EW, Tamminga R, van der Hoeven H (2013). Preoperative patient and injury factors of successful rehabilitation after anterior cruciate ligament reconstruction with single-bundle techniques. Arthroscopy.

[13] Ding DY, Chang RN, Allahabadi S, Coughlan MJ, Prentice HA, Maletis GB (2022). Acute and subacute anterior cruciate ligament reconstructions are associated with a higher risk of revision and reoperation. Knee Surg Sports Traumatol Arthrosc.

[14] Erard J, Cance N, Shatrov J, Fournier G, Gunst S, Ciolli G (2023). Delaying ACL reconstruction is associated with increased rates of medial meniscal tear. Knee Surg Sports Traumatol Arthrosc.

[15] Eriksson K, von Essen C, Jönhagen S, Barenius B (2018). No risk of arthrofibrosis after acute anterior cruciate ligament reconstruction. Knee Surg Sports Traumatol Arthrosc.

[16] Faunø P, Rahr-Wagner L, Lind M (2014). Risk for revision after anterior cruciate ligament reconstruction is higher among adolescents: results from the Danish registry of knee ligament reconstruction. Orthop J Sports Med.

[17] Ferguson D, Palmer A, Khan S, Oduoza U, Atkinson H (2019). Early or delayed anterior cruciate ligament reconstruction: Is one superior? A systematic review and meta-analysis. Eur J Orthop Surg Traumatol.

[18] Frobell RB, Roos EM, Roos HP, Ranstam J, Lohmander LS (2010). A randomized trial of treatment for acute anterior cruciate ligament tears. N Engl J Med.

[19] Fältström A, Hägglund M, Magnusson H, Forssblad M, Kvist J (2016). Predictors for additional anterior cruciate ligament reconstruction: data from the Swedish national ACL register. Knee Surg Sports Traumatol Arthrosc.

[20] Grindem H, Snyder-Mackler L, Moksnes H, Engebretsen L, Risberg MA (2016). Simple decision rules can reduce reinjury risk by 84% after ACL reconstruction: the Delaware-Oslo ACL cohort study. Br J Sports Med.

[21] Herbst E, Hoser C, Gföller P, Hepperger C, Abermann E, Neumayer K (2017). Impact of surgical timing on the outcome of anterior cruciate ligament reconstruction. Knee Surg Sports Traumatol Arthrosc.

[22] Kaeding CC, Pedroza AD, Reinke EK, Huston LJ, Spindler KP (2015). Risk factors and predictors of subsequent ACL injury in either knee after acl reconstruction: prospective analysis of 2488 primary ACL reconstructions from the MOON cohort. Am J Sports Med.

[23] Kim SH, Han SJ, Park YB, Kim DH, Lee HJ, Pujol N (2021). A systematic review comparing the results of early vs delayed ligament surgeries in single anterior cruciate ligament and multiligament knee injuries. Knee Surg Relat Res.

[24] Kopf S, Kauert R, Halfpaap J, Jung T, Becker R (2012). A new quantitative method for pivot shift grading. Knee Surg Sports Traumatol Arthrosc.

[25] Krutsch W, Zellner J, Baumann F, Pfeifer C, Nerlich M, Angele P (2017). Timing of anterior cruciate ligament reconstruction within the first year after trauma and its influence on treatment of cartilage and meniscus pathology. Knee Surg Sports Traumatol Arthrosc.

[26] Kwok CS, Harrison T, Servant C (2013). The optimal timing for anterior cruciate ligament reconstruction with respect to the risk of postoperative stiffness. Arthroscopy.

[27] Lee YS, Lee OS, Lee SH, Hui TS (2018). Effect of the timing of anterior cruciate ligament reconstruction on clinical and stability outcomes: a systematic review and meta-analysis. Arthroscopy.

[28] Lind M, Menhert F, Pedersen AB (2009). The first results from the Danish ACL reconstruction registry: epidemiologic and 2 year follow-up results from 5,818 knee ligament reconstructions. Knee Surg Sports Traumatol Arthrosc.

[29] Lunde AS, Lundeborg S, Lettenstrom GS, Thygesen L, Huebner J (1980). The person-number systems of Sweden, Norway, Denmark, and Israel. Vital Health Stat.

[30] Magnussen R, Reinke EK, Huston LJ, Andrish JT, Cox CL, Dunn WR (2019). Anterior and rotational knee laxity does not affect patient-reported knee function 2 years after anterior cruciate ligament reconstruction. Am J Sports Med.

[31] Maletis GB, Chen J, Inacio MC, Funahashi TT (2016). Age-related risk factors for revision anterior cruciate ligament reconstruction: a cohort study of 21,304 patients from the kaiser permanente anterior cruciate ligament registry. Am J Sports Med.

[32] Mather RC, Hettrich CM, Dunn WR, Cole BJ, Bach BR, Huston LJ (2014). Cost-effectiveness analysis of early reconstruction versus rehabilitation and delayed reconstruction for anterior cruciate ligament tears. Am J Sports Med.

[33] Passler JM, Schippinger G, Schweighofer F, Fellinger M, Seibert FJ (1995). Complications in 283 cruciate ligament replacement operations with free patellar tendon transplantation. modification by surgical technique and surgery timing. Unfallchirurgie.

[34] Prodromidis AD, Drosatou C, Mourikis A, Sutton PM, Charalambous CP (2022). Relationship between timing of anterior cruciate ligament reconstruction and chondral injuries: a systematic review and meta-analysis. Am J Sports Med.

[35] Rahardja R, Zhu M, Love H, Clatworthy MG, Monk AP, Young SW (2020). Rates of revision and surgeon-reported graft rupture following ACL reconstruction: early results from the New Zealand ACL Registry. Knee Surg Sports Traumatol Arthrosc.

[36] Rahr-Wagner L, Thillemann TM, Lind MC, Pedersen AB (2013). Validation of 14,500 operated knees registered in the Danish Knee Ligament Reconstruction Register: registration completeness and validity of key variables. Clin Epidemiol.

[37] Roos EM, Toksvig-Larsen S (2003). Knee injury and osteoarthritis outcome score (KOOS)—validation and comparison to the WOMAC in total knee replacement. Health Qual Life Outcomes.

[38] Runer A, Csapo R, Hepperger C, Herbort M, Hoser C, Fink C (2020). Anterior cruciate ligament reconstructions with quadriceps tendon autograft result in lower graft rupture rates but similar patient-reported outcomes as compared with hamstring tendon autograft: a comparison of 875 patients. Am J Sports Med.

[39] Samuelsen BT, Webster KE, Johnson NR, Hewett TE, Krych AJ (2017). Hamstring autograft versus patellar tendon autograft for acl reconstruction: is there a difference in graft failure rate? A meta-analysis of 47,613 Patients. Clin Orthop Relat Res.

[40] Shelbourne KD, Wilckens JH, Mollabashy A, DeCarlo M (1991). Arthrofibrosis in acute anterior cruciate ligament reconstruction. The effect of timing of reconstruction and rehabilitation. Am J Sports Med.

[41] Shen X, Liu T, Xu S, Chen B, Tang X, Xiao J (2022). Optimal timing of anterior cruciate ligament reconstruction in patients with anterior cruciate ligament tear: a systematic review and meta-analysis. JAMA Netw Open.

[42] Snaebjörnsson T, Hamrin Senorski E, Svantesson E, Westin O, Persson A, Karlsson J (2019). Graft fixation and timing of surgery are predictors of early anterior cruciate ligament revision: a cohort study from the swedish and norwegian knee ligament registries based on 18,425 patients. JB JS Open Access.

[43] Tegner Y, Lysholm J (1985). Rating systems in the evaluation of knee ligament injuries. Clin Orthop Relat Res.

[44] Vermeijden HD, Yang XA, Rademakers MV, Kerkhoffs G, van der List JP, DiFelice GS (2022). Early and delayed surgery for isolated ACL and multiligamentous knee injuries have equivalent results: a systematic review and meta-analysis. Am J Sports Med.

[45] von Essen C, Eriksson K, Barenius B (2020). Acute ACL reconstruction shows superior clinical results and can be performed safely without an increased risk of developing arthrofibrosis. Knee Surg Sports Traumatol Arthrosc.

[46] Webster KE, Feller JA, Leigh WB, Richmond AK (2014). Younger patients are at increased risk for graft rupture and contralateral injury after anterior cruciate ligament reconstruction. Am J Sports Med.

[47] Wiggins AJ, Grandhi RK, Schneider DK, Stanfield D, Webster KE, Myer GD (2016). Risk of secondary injury in younger athletes after anterior cruciate ligament reconstruction: a systematic review and meta-analysis. Am J Sports Med.

[48] Yabroudi MA, Björnsson H, Lynch AD, Muller B, Samuelsson K, Tarabichi M (2016). Predictors of Revision surgery after primary anterior cruciate ligament reconstruction. Orthop J Sports Med.

